# Comprehensive analysis of the *Corynebacterium glutamicum* transcriptome using an improved RNAseq technique

**DOI:** 10.1186/1471-2164-14-888

**Published:** 2013-12-17

**Authors:** Katharina Pfeifer-Sancar, Almut Mentz, Christian Rückert, Jörn Kalinowski

**Affiliations:** 1 Microbial Genomics and Biotechnology, Center for Biotechnology, Bielefeld University, Universitätsstraße 27, 33615, Bielefeld, Germany; 2 Technology Platform Genomics, Center for Biotechnology, Bielefeld University, Universitätsstraße 27, 33615, Bielefeld, Germany

**Keywords:** *Corynebacterium glutamicum*, RNA, High-throughput sequencing, Transcriptome

## Abstract

**Background:**

The use of RNAseq to resolve the transcriptional organization of an organism was established in recent years and also showed the complexity and dynamics of bacterial transcriptomes. The aim of this study was to comprehensively investigate the transcriptome of the industrially relevant amino acid producer and model organism *Corynebacterium glutamicum* by RNAseq in order to improve its genome annotation and to describe important features for transcription and translation.

**Results:**

RNAseq data sets were obtained by two methods, one that focuses on 5′-ends of primary transcripts and another that provides the overall transcriptome with an improved resolution of 3′-ends of transcripts. Subsequent data analysis led to the identification of more than 2,000 transcription start sites (TSSs), the definition of 5′-UTRs (untranslated regions) for annotated protein-coding genes, operon structures and many novel transcripts located between or in antisense orientation to protein-coding regions. Interestingly, a high number of mRNAs (33%) is transcribed as leaderless transcripts. From the data, consensus promoter and ribosome binding site (RBS) motifs were identified and it was shown that the majority of genes in *C. glutamicum* are transcribed monocistronically, but operons containing up to 16 genes are also present.

**Conclusions:**

The comprehensive transcriptome map of *C. glutamicum* established in this study represents a major step forward towards a complete definition of genetic elements (e.g. promoter regions, gene starts and stops, 5′-UTRs, RBSs, transcript starts and ends) and provides the ideal basis for further analyses on transcriptional regulatory networks in this organism. The methods developed are easily applicable for other bacteria and have the potential to be used also for quantification of transcriptomes, replacing microarrays in the near future.

## Background

*Corynebacterium glutamicum* is a non-pathogenic, non-sporulating, gram-positive soil bacterium that belongs to the order *Actinomycetales*. This microorganism is widely used for the production of various amino acids and other industrially relevant compounds [1,2]. Furthermore, the availability of genetic engineering methods, an easy cultivation and a generally-regarded-as-safe status has helped to make it a model organism for systems biology investigations in the *Corynebacterineae*, comprising important pathogens such as *Corynebacterium diphtheriae* and *Mycobacterium tuberculosis*[3]*.* The genome sequence of the 3.3 Mb circular chromosome was established a decade ago [4,5] and contains more than 3,000 annotated protein-coding sequences (CDS). Based on the complete genome sequence, transcriptional regulation in *C. glutamicum* has been studied extensively [6] and revealed a complex regulatory network including 97 transcriptional regulator proteins with so far 1,432 regulatory interactions [7]. In addition, *C. glutamicum* possesses seven sigma factors regulating transcription on a global scale, recognizing specific promoter signals [8]. Although the promoters of about 200 genes have been identified in the last two decades, this leaves the majority of genes without known transcription signals. Recently, small RNAs (sRNA) were studied in *C. glutamicum* on a global scale, demonstrating the usefulness of the recently developed method of high-throughput sequencing of cDNA (RNAseq) [9].

The understanding and deciphering of transcriptome complexity of an organism and the underlying functionalities have become a major focus for post-genome research in recent years [10,11]_._ Beside the classical approaches for profiling transcripts like Northern blots, reverse-transcriptase (q)PCR, RACE (rapid amplification of cDNA ends), and microarrays, the recent development of RNAseq has revolutionized transcriptomics. This allows to analyze transcriptomes not only in a completely comprehensive way but also with single-nucleotide resolution [12,13]. The features of RNAseq that are unmatched by the classical approaches, i.e. no background or saturation effects as in fluorescence-based detection, no cross-hybridization, and therefore an almost unrestricted dynamic range of detection, make RNAseq an attractive approach to analyze the entire transcriptome also quantitatively [12].

This novel sequencing approach has been successfully applied for studying whole-genome transcription for various prokaryotes and eukaryotes [14-20] and revealed an unexpected complexity of these transcriptomes, e.g. widespread antisense transcription and an enormous amount of small and novel RNAs in bacterial genomes [12,21-24]. Additionally, this method offers the opportunity to improve genome annotation for prokaryotes and eukaryotes, in the latter especially regarding exon identification and alternative splicing effects [12,25,26].

Beside the use of RNAseq results mapping of complete transcripts, detection of transcription start sites and the analysis of the respective promoters, the transcriptome data can be further analyzed to characterize RBSs, providing important information also on translational processes.

Quite often 5′-UTRs have sizes up to several hundred bases, indicating more complex transcriptional and translational functions such as riboswitches, RNA thermometers or binding sites for regulatory RNAs [14,15,21,22]. In contrast, RNAseq analyses have demonstrated that some transcripts are leaderless, imposing a different translation mechanism [14,15].

Further important information obtainable from RNAseq data is the arrangement of genes in operons. The classical operon has multiple genes which are transcribed from a single promoter [27,28]. Operons typically contain genes that are functionally connected, e.g. in a metabolic pathway [29,30]. Hence, this feature might be helpful in prediction of gene function. Moreover, recent RNAseq data showed that various operons have to be divided into sub-operons due to internal transcription start sites which often respond to different conditions [15,21]. Thus, operon structures are not always simple, but can have rather complex architectures that can be fully resolved by RNAseq analysis.

The RNAseq workflow is complex and a number of technical obstacles have to be overcome. First, the majority of total RNA in a bacterial cell consists of ribosomal RNA (> 95% rRNA). This rRNA has to be removed efficiently either by hybridization-based rRNA depletion or enzyme-based degradation of processed transcripts (including rRNA) [22]. Second, it is important to maintain the strand information to be able to discriminate between sense and antisense transcripts. Methods allowing to obtain this information have been developed for eukaryotes [19,31], and were also adapted for prokaryotes [15,32].

For bacterial RNAseq studies Illumina, 454 and SOLiD sequencing platforms have been used [14,15,17,26,33]. For RNAseq analyses of organisms with known genome sequence, a high number of short reads (20 – 50 nt) is preferable to a small number of long reads, at least for microbial genomes.

In this study we describe an improved RNAseq method that provides a strand-specific characterization of entire transcriptomes at a whole genome level using high-throughput sequencing. Furthermore, we developed two RNAseq library preparation protocols that allow for analyzing the primary transcriptome and the total transcriptome of an organism separately. Additionally, we applied these two RNAseq protocols successfully to the transcriptome of *C. glutamicum.* By sequencing the primary transcriptome, we utilized RNA samples from exponential growth phase to analyze TSSs recognized by the housekeeping sigma factor σ^A^. The investigation of the obtained RNAseq data delivered more than 2,000 TSSs which helped to correct more than 200 gene starts and the detection of a quite high number of leaderless transcripts (> 700). For sequencing the whole transcriptome we used RNA samples from nine different conditions (exponential growth phase, heat and cold shock, salt stress, oxidative stresses, and ethanol stress) to obtain a broad range of transcripts. The analysis of this data leads to the identification of operon structures and the detection of novel transcripts in *C. glutamicum*.

## Results

### Development of native 5′-end and whole transcript RNAseq protocols

**Figure 1:**
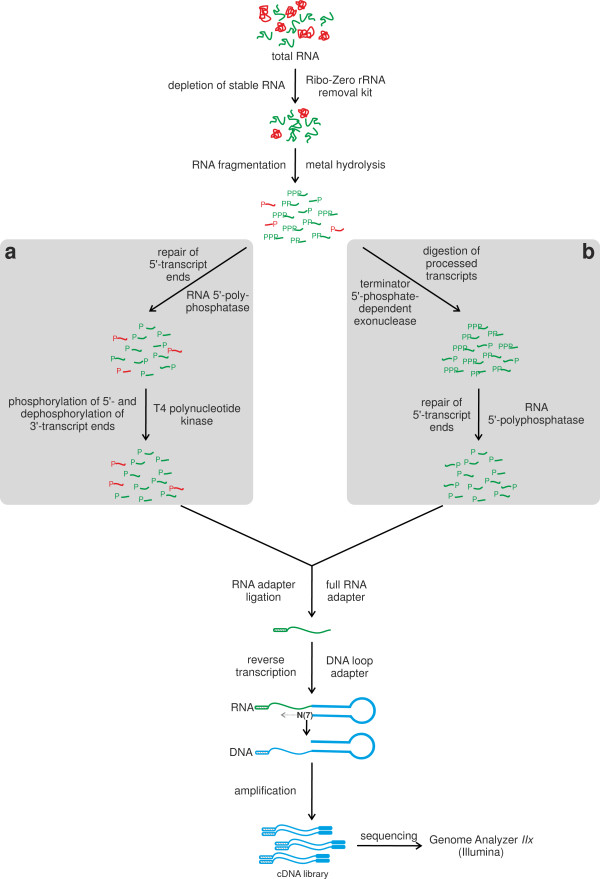
**Experimental workflow for the preparation of a whole transcriptome library (a) and of a library enriched for primary 5′-transcript ends (b).** Both protocols start with isolated total RNA. Stable RNA is then depleted using the Ribo-Zero rRNA removal kit and the obtained RNA is fragmented my metal hydrolysis to a size of 200 - 500 nt. For the whole transcriptome library **(a)** the 5′-triphosphate ends are processed to 5′-monophosphate ends by a RNA 5′-polyphosphatase, unphosphorylated 5′-ends are phosphorylated, and phosphorylated 3′-ends are then dephosphorylated using T4 polynucleotide kinase. For the native 5′-end protocol **(b)**, all fragments containing a 5′-monophosphate are degraded by treatment with a 5′-phosphate dependent exonuclease and the 5′-triphosphate ends of native transcripts are then processed to 5′-monophosphate ends by a RNA 5′-polyphosphatase. Next, for both libraries RNA adapters are ligated to the 5′-ends carrying a 5′-monophosphate group. The tagging of the 3′-end of the RNA with flanking sequences necessary for reverse transcription is performed in a ligation-free approach with a loop DNA adapter containing seven unpaired wobble bases at its 3′-end. After reverse transcription of the RNA fragments into cDNA fragments, the cDNA fragments are amplified, tagged with sequencing linkers at their ends by PCR and finally sequenced. Stable RNA species (rRNA, tRNA) are depicted in red, other RNAs are given in green, and DNA in blue.

To analyze the whole transcriptome as well as the native transcription start sites of *C. glutamicum*, i.e. those that originate from initiation of transcription by RNA polymerase, a whole transcriptome RNAseq protocol (Figure 1a) and a native 5′-end RNAseq protocol were developed (Figure 1b), adapting the differential RNA-seq approach [15]. Key differences of our protocol compared to the differential RNA-seq method include:

I) Depletion of stable RNAs using the Ribo-Zero rRNA removal kit, as preliminary tests indicated that rRNA constitutes up to 99% of the total RNA in *C. glutamicum* (data not shown), necessitating an efficient removal in both libraries.

II) RNA fragmentation by metal hydrolysis to allow for more efficient ligation of the 5′-end RNA adapter and better accessibility for the RNA loop adapter. This should result in an increase of completely reverse transcribed cDNAs carrying both adapter sequences needed for high-throughput sequencing. In case of the native 5′-end protocol, this also causes only fragments containing either a 5′-triphosphate (start of a native transcript) or an unphosphorylated 5′-end (resulting from metal hydrolysis) to be retained upon Terminator 5′-phosphate-dependent exonuclease.

III) Repair of the 5′-ends of the RNA fragments to enable ligation of the 5′-end RNA adapter. Both protocols apply an RNA 5′-polyphosphatase treatment converting the 5′-triphosphates of primary transcript ends to monophosphates to allow ligation. Thus in case of the native 5′-end protocol, only fragments derived from a 5′-end of a primary transcript will retain a 5′-monophosphate. In case of the whole transcriptome protocol, an additional treatment with T4 polynucleotide kinase is performed, phosphorylating all 5′-ends lacking a phosphate group and thus enhancing adapter ligation efficiency.

IV) For cDNA synthesis, a loop adapter is used which hybridizes via a 3′-NNNNNNN-tail preferentially to the 3′-end of the RNA fragments as the binding there is stabilized by the stacking energy of the formed DNA-RNA duplex. This approach saves a preparation step for the reverse transcription (e.g., polyA-tailing, ligation of a 3′-adapter) and allows the direct accessibility of the 3′-fragment ends as the sequence used in the stem of the loop adapter is identical to the Illumina Paired-Read Primer 2.

V) Several size selection steps are performed to remove adapter dimers.

VI) Sequencing is done using Illumina technology, allowing for a much deeper sequencing and the possibility to obtain paired-end information

The whole transcriptome RNAseq protocol was developed to determine operon structures, so far unknown transcripts as well as transcript ends. During this procedure the primary transcripts are not enriched, enabling the sequencing of processed and native transcripts. Moreover, the whole transcriptome protocol enables read coverage of entire transcripts, including their 3′-ends. As this approach should deliver expression of as many transcripts as possible, the RNA samples for this data set originated from mixed *C. glutamicum* cell samples grown under different cultivation conditions (minimal or complex media w/o stress application, and minimal medium but stressed with 10% ethanol, 10% sodium chloride, 90% dissolved oxygen, 1% hydrogen peroxide, 2 mM diamide, heat stress at 50°C as well as cold stress at 4°C).

The objective of generating the native 5′-end data set was to identify as many TSSs as possible and to localize promoters, RBSs and 5′-UTRs of *C. glutamicum* in a comprehensive way. We were mainly interested in TSSs recognized by the housekeeping sigma factor σ^A^. Therefore the RNA samples for this data set originated from mixed *C. glutamicum* cell samples grown in minimal and in complex media. The mixing of cells from both conditions should ensure transcription of most housekeeping genes, including those involved in anabolism and in catabolism.

### Data generation by Illumina sequencing and mapping of DNA sequence reads to the *Corynebacterium glutamicum* ATCC13032 genome

In order to characterize the primary and the whole transcriptome of *C. glutamicum* using RNAseq, the two described RNAseq libraries were sequenced on a Genome Analyzer *II*x (Illumina). A total of 2 × 20.53 and 20.76 million reads were generated from the whole and the primary transcriptome library, respectively. These reads of 26 nt in length (one low quality base trimmed from the 3′-end of the sequenced 27 nt reads) were mapped to the *C. glutamicum* genome. After mapping of reads and removal of duplicate mappings (i.e. reads mapping to repeat regions like rRNAs), 15.96 and 2.65 million reads were mapped uniquely to the genome from the whole and the primary transcriptome library, respectively (Table 1).

**Table 1:** Summary of sequencing and mapping statistics for the whole transcriptome and primary 5′-transcript ends library

	**whole transcriptome**^**a**^	**primary 5′-transcript ends**^**a**^
total reads	2 × 20.53	20.76
total mappings^b^	22.19	5.07
total mappings of CPR^c^	13.91	-
reads mapping at multiple positions^b^	1.19	0.68
CPR mapping at multiple positions	0.72	-
reads mapping at a single position^b^	15.96	2.65
CPR mapping at a single position	10.20	-

^a^in million.

^b^in case of the whole transcriptome data: sum of combined read pairs, forward reads without reverse mates, and reverse reads without forward mates that map to the genome sequence of *C. glutamicum*; in case of primary 5′-transcript ends data: forward reads that map to the genome sequence of *C. glutamicum.*

^c^CPR: combined pair of reads.

### Comprehensive identification of transcription start sites from the native 5′-end data set

**Figure 2:**
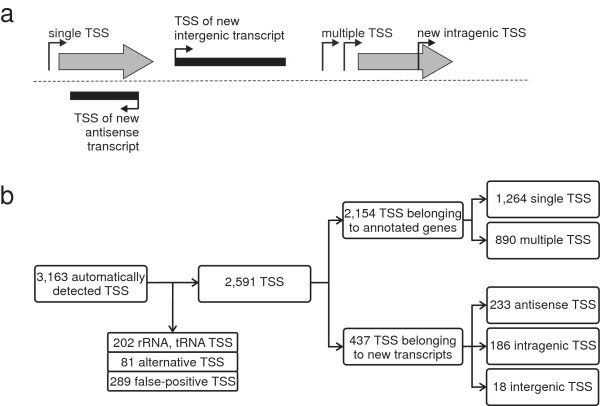
**Classification of TSSs obtained with RNAseq. (a)** Illustration of categories for TSS classification based on genomic context. The first TSS classification level is divided into two categories: TSSs that belong to annotated genes (gray shaded arrows) and TSSs that belong to new transcripts (black shaded arrows). TSSs belonging to annotated genes were classified into single TSSs or multiple TSSs. TSSs belonging to new transcripts were arranged into antisense, intragenic or intergenic TSSs. **(b)** Identification, filtering, and classification of TSSs. From the automatically detected TSSs those TSSs were removed that belong to rRNA or tRNA, false-positive, or alternative TSSs.

The analysis of the native 5′-end data set resulted in the identification of 3,163 TSSs. This number also contains alternative TSSs that originate from one promoter and such that belong to rRNA and tRNA genes. The total number of TSSs was reduced by merging alternative TSSs (81) and removing TSSs that belong to rRNA and tRNA genes (202) or false-positive TSSs (289). An alternative transcription start was assumed to be in a range of ≤ 1 base. The +1 position with the highest number of read starts within this range was selected as transcription start. After the elimination of alternative, redundant rRNA and tRNA, and false-positive signals, 2,591 TSSs remain (Figure 2; Additional file 1: Table S1).

These TSSs can be classified into two groups: TSSs which belong to annotated genes and TSSs that are assigned to novel transcripts. TSSs belonging to annotated genes were further categorized into 1,264 single TSSs (a single TSS per gene) and 890 multiple TSSs (more than one TSS per gene), the latter occurring at 365 genes. TSS numbers per gene could be as high as six, e.g. as found for *cmt1*, which encodes a trehalose corynomycolyl transferase (Additional file 2: Figure S1).

TSSs belonging to novel transcripts were classified into three categories: antisense TSSs – the respective transcript is allocated in antisense orientation to an annotated gene; intragenic TSSs – these TSSs are located within annotated genes in sense orientation; intergenic TSSs – these TSSs are located between annotated genes (Figure 2).

In total, 2,154 TSS were assigned to annotated genes, whereby 2,147 of 2,154 belong to genes encoding proteins and the remaining seven were assigned to non-coding RNA (one TSS belonging to 4.5S RNA, M1 RNA, and 6C RNA genes, respectively, as well as four TSSs belonging to tmRNA). In summary, for 1,629 of the 3,043 (53.5%) actually annotated genes in *C. glutamicum*[34] a TSS was found. Additionally, 437 TSSs could not be assigned to annotated genes, but to novel, so far unknown transcripts. Overall, 233 of these 437 TSSs belong to antisense transcripts, 186 relate to intragenic transcripts and 18 are assigned to new intergenic transcripts (Figure 2).

### Identification of σ^A^-dependent promoters

Promoter motifs are sites on the DNA to which the RNA polymerase attaches in order to start transcription [35,36]. In bacteria, different species of sigma factors have been identified that are components of the RNA polymerase holoenzyme, each recognizing a different promoter motif and thus contributing to transcription of a particular set of genes [37-39]. The major sigma factor, σ^70^, has been found in all known bacterial species and is responsible for the transcription of housekeeping genes [8,37,38]. σ^70^ binds to specific promoter elements, called the -10 and a -35 region [8,39-42].

**Figure 3:**
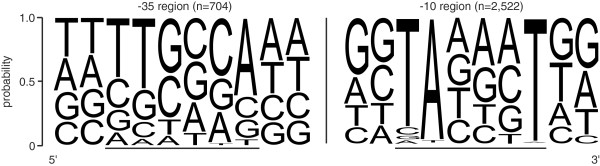
**Distribution of nucleotides within the -10 and -35 regions of *C. glutamicum *σ**^**A **^**promoters.** Relative occurrence of a nucleotide at a particular position is represented by the size of the nucleotide. The representation is based on 2,522 -10 and 704 -35 regions identified with *Improbizer*[43]. The core -10 and -35 regions are underlined. The sequence logo was created with *WebLogo*[45].

Thus, using the identified TSSs, it is possible to search for promoter motifs*.* For this search the web program *Improbizer*[43] was used to scan 60 bases upstream of each of the 2,591 identified TSSs. The conserved -10 motif “TAnnnT” was found in about 97% (2,522 in total) of the upstream sequences of the identified TSSs with a spacer (distance between -10 motif and TSS) of 3 - 11 nt (Figure 3, Additional file 1: Table S1). The motif as well as the distance of the motif to the TSS are similar to the published σ^A^ consensus promoter of *C. glutamicum*[8,44].

Then, a -35 region was searched using the 2,522 upstream sequences with identified -10 motifs. As a further requirement for the identification of the -35 motif the position of the found motif was taken into account. Therefore, only a spacer length (distance between the -10 and -35 region) of 16 - 19 nt was allowed [8]. Regarding these requirements 704 motifs could be determined showing a weakly conserved “ttgnca” motif (Figure 3, Additional file 1: Table S1).

This motif fits the previously published data for *C. glutamicum*[8,44]*.*

### Re-annotation of coding sequences

The RNAseq data containing the TSSs turned out to be very useful for the correction of translational starts of coding sequence. Especially leaderless mRNAs were conducive for re-annotation. In total, the translational start codon positions of 205 genes were corrected. As a result of this re-annotation, 185 genes now encode leaderless mRNAs and the remaining 20 are genes containing 5′-UTRs (Additional file 1: Table S2). The TSSs of these leadered genes mapped within the particular gene but not on a start codon that is in-frame to the annotated stop codon, so that a leaderless mRNA for the appropriate gene could be excluded and a new, corrected translational start codon was searched downstream of the TSS. For this search, DNA sequences from the TSS position to the annotated stop codon of each of the 20 genes were scanned using the web program *ORF finder*[46] with ATG, GTG, CTG, and TTG as start codons and TGA, TAA, and TAG as stop codons. The identified and most meaningful gene starts for the 20 genes containing 5′-UTRs are listed in Additional file 1: Table S2.

For further RNAseq analyses, especially for the determination of 5′-UTRs, analysis of RBSs, identification of operons structures, and detection of novel transcripts, these re-annotated gene starts were used.

### Characteristics of 5′-UTRs and leaderless mRNAs

The 2,147 TSS that were assigned to annotated, protein coding sequences were used for an analysis of the 5′-UTRs, the part of a transcript reaching from the TSS to the start codon. Quite surprisingly, ~33% (707 of 2,147) of the mRNAs in *C. glutamicum* were found to have no 5′-UTR (5′-UTR length = 0; Additional file 1: Table S3). These mRNAs were classified as leaderless in this study. This high fraction of leaderless mRNA do not show any significant preference to functional *eggNOG*[47] categories (data not shown). The analysis of initiator codons of such mRNAs in *C. glutamicum* revealed that all leaderless mRNAs have an AUG (~79%) or GUG (~21%) start codon.

Beside the many leaderless genes, there is a further accumulation of 5′-UTRs with lengths between 26 - 40 nt (278 in total). These short leaders might only harbor a ribosome-binding site for translation.

**Figure 4:**
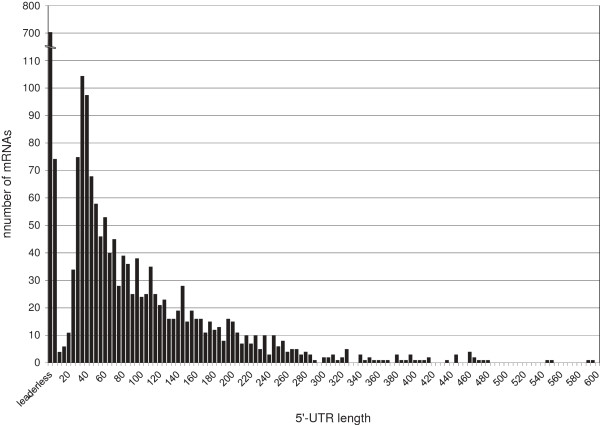
**Distribution of 5′-UTR length of mRNAs belonging to annotated protein-coding genes in *C. glutamicum.*** The distribution is based on 2,147 TSSs assigned to mRNAs. The bar labeled leaderless represents an UTR length of zero. The other bars represent UTR length in increments of five (1 - 5, 6 - 10, 11 - 15, *etc.*).

Analysis of the distribution of the 5′-UTR lengths revealed that in general the number of 5′-UTRs within a certain bin decreased exponentially with increasing length (Figure 4). However, an interesting exception is represented by 80 5′-UTRs with a length of 1 - 10 nt. These 5′-UTRs do not provide enough sequence for a RBS and spacing to the initiation codon. Hence, the mechanism of translation initiation for such mRNAs and whether such transcripts result in functional proteins remains unclear.

A relatively high number of mRNAs contain 5′-UTRs longer than 100 nt (531 of 2,147 in total; Additional file 1: Table S3). It can be assumed that these long 5′-UTRs play a role in the translational regulation of their respective mRNAs through folding into secondary structures. Such *cis*-regulatory mechanisms of 5′-UTRs were previously described for many bacteria and can harbor sequences encoding leader peptides, riboswitches, RNA thermometers or binding sites for *trans*-encoded RNAs [13,48,49].

To detect putative *cis*-regulatory 5′-UTR candidates, *Rfam*[50] database predicted regulatory regions within 5′-UTRs in *C. glutamicum* were compared with the RNAseq data obtained. Altogether, 16 regulatory regions were predicted for *C. glutamicum* and 13 of them matched the RNAseq data (Table 2). Supporting evidence for these riboswitches was provided by the annotation of the associated protein-coding regions. For example, the gene *cg0083* encodes a predicted mononucleotide transporter and shows a putative flavin mononucleotide (FMN) riboswitch within the 5′-UTR for sensing flavin mono- and/or dinucleotides. Furthermore, the products of the genes *cg1476* (*thiC*)*, cg1655* (*thiM*), and *cg2236* (*thiE*) are involved in thiamine metabolism and these genes contain putative thiamine pyrophosphate (TPP)-sensing riboswitches within their 5′-UTRs. It is attractive to speculate that the two other genes with a predicted and validated TPP riboswitch, *cg0825* encoding a putative short-chain oxidoreductase and *cg1227*, encoding part of an ABC transporter, are also involved in thiamine-dependent processes*.* Additionally, one RNA thermometer, the *cspA* mRNA 5′-UTR, was observed within the 5′-UTR of *cg0215* that encodes a predicted cold-shock protein.

**Table 2:** ***Rfam *** **predictions for regulatory regions in *C. glutamicum *** **compared to RNAseq data**

***Rfam *** **prediction**^**a**^						**RNAseq identification**^**b**^			
**Name**	**ID**	**Start**	**End**	**Bit Score**	**Strand**	**RNAseq**	**Start**	**End**	**Gene**
FMN riboswitch	RF00050	66,442	66,279	111.60	-	observed	66,438	66,198	*cg0083*
cspA mRNA 5′-UTR	RF01766	186,399	186,766	60.19	+	observed	186,328	186,508	*cg0215 (cspA)*
TPP riboswitch	RF00059	742,654	742,547	63.54	-	observed	742,651	742,490	*cg0825*
ydaO-yuaA leader	RF00379	870,027	869,859	69.93	-	observed	870,047	869,853	*cg0936 (rpf1)*
TPP riboswitch	RF00059	1,127,774	1,127,883	51.03	+	observed	1,127,765	1,127,874	*cg1227*
mini-ykkC RNA motif	RF01068	1,131,047	1,131,094	33.52	+	not observed	-	-	*-*
TPP riboswitch	RF00059	1,373,213	1,373,103	55.87	-	observed	1,373,210	1,373,105	*cg1476 (thiC)*
SAM-IV riboswitch	RF00634	1,374,007	1,374,123	70.47	+	observed	1,374,005	1,374,139	*cg1478*
TPP riboswitch	RF00059	1,544,490	1,544,383	52.11	-	observed	1,544,485	1,544,390	*cg1655 (thiM)*
yybP-ykoY leader	RF00080	1,550,030	1,550,196	43.71	+	not observed	-	-	*-*
yybP-ykoY leader	RF00080	2,043,157	2,042,989	49.13	-	observed	2,043,151	2,042,955	*cg2157 (terC)*
TPP riboswitch	RF00059	2,120,271	2,120,383	62.55	+	observed	2,120,271	2,120,384	*cg2236 (thiE)*
mraW RNA motif	RF01746	2,267,021	2,266,916	56.64	-	observed	2,266,932	2,266,800	*cg2377 (mraW)*
ydaO-yuaA leader	RF00379	2,292,467	2,292,279	59.37	-	observed	2,292,509	2,292,267	*cg2402*
msiK RNA motif	RF01747	2,582,375	2,582,317	52.13	-	observed	2,582,404	2,582,315	*cg2708 (msiK1)*
yybP-ykoY leader	RF00080	2,649,004	2,648,890	49.90	-	not observed	-	-	*-*

^a^Name, ID, coordinates, and bit score of predicted regulatory 5′-UTRs for *C. glutamicum* were taken from the *Rfam* database [50].

^b^Refers to detected TSSs and RNAseq ends relate to the last position of the 5′-UTR. The secondary structures of the verified regulatory regions are shown in Additional file 3: Figure S2.

To identify possible secondary structures within the regulatory 5′-UTRs predicted by *Rfam*[50] and validated by RNAseq, the web server program *RNAfold*[51] was utilized. The sequences of the putative regulatory 5′-UTRs including 15 bases of the coding region were used for the structure predictions. In all 13 cases, the 5′-UTRs can fold into stable stem-loop structures, where the RBS is (partly) sequestered (Additional file 3: Figure S2).

It should also be mentioned here that small leader peptides within 5′-UTRs of genes and operons involved in amino acid synthesis (*cg3359/trpE*, *cg0303/leuA*, *cg1435/ilvB*, and *cg1129/aroF*) were detected in *C. glutamicum* by RNAseq analysis [9]. These leader peptides are possibly involved in transcription attenuation and control the expression of the appropriated genes dependent on the amino acid level [52,53].

### Analysis of ribosome binding sites

**Figure 5:**
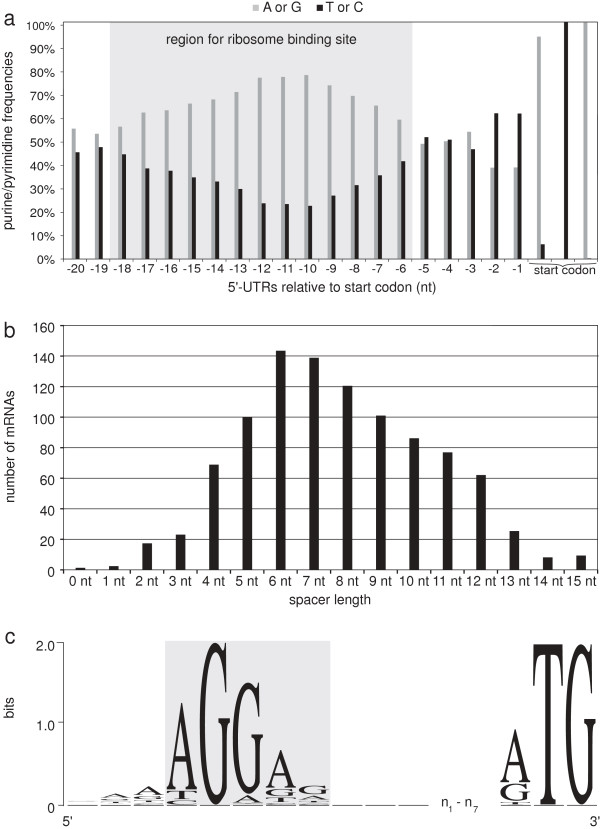
**Analysis of ribosome binding sites in *C. glutamicum. *(a)** Frequencies of purines (G or A) compared to frequencies of pyrimidines (T or C) within the first 20 bases (relative to the start codon) of 922 different 5′-UTRs. **(b)** Analysis of the spacing between the RBS and the start codon, based on all identified RBS motifs by *Improbizer*. **(c)** Information content within the identified RBS motif of *C. glutamicum* including three leading and lagging bases, a spacer of 1 - 7 nt, as well as the translational initiation codon*.* The y-axis shows the information content (measured in bits). The analysis is based on 673 RBS motifs identified with *Improbizer* within a spacing of 7 ± 3 nt only. The sequence logo was created with *WebLogo*[45].

Based on the 5′-UTRs of protein-coding genes, it is possible to analyze RBSs. Therefore, the frequency of purines (G and A) compared to that of pyrimidines (T and C) of each nucleotide within the 20 nt upstream of the start codon was computed (Figure 5a) and showed an accumulation of purines (> 55%) in the region between 6 to 18 bp upstream of the start codon with a peak around -11 relative to the first base of the start codon (Figure 5a) indicating a RBS within this region. A scan for a sequence motif within this region (14 – 20 bases upstream of the initiator codon; 928 sequences in total) using the web program *Improbizer*[43] confirmed this indication by detecting the conserved motif AGGag in about 92% of the 5′-UTR sequences (Figure 5c, Additional file 1: Table S4).

Next, the distance distribution between the RBS and the initiator codon was calculated by binning the length of the spacers. The distribution for all detected RBS revealed a spacing of 4 - 12 nt as being the most common (> 90%), with 7.7 ± 2.7 nt as mean spacing (Figure 5b).

Here, we analyzed only ribosome sites that are located in the 5′-UTR of transcripts. RBSs within intercistronic regions were excluded from this analysis and will be addressed in more detail in future studies. However, the identified RBS motif AGGag can also be found within those intercistronic regions (data not shown).

### Identification of operon structures based on the whole transcriptome data set

**Figure 6:**
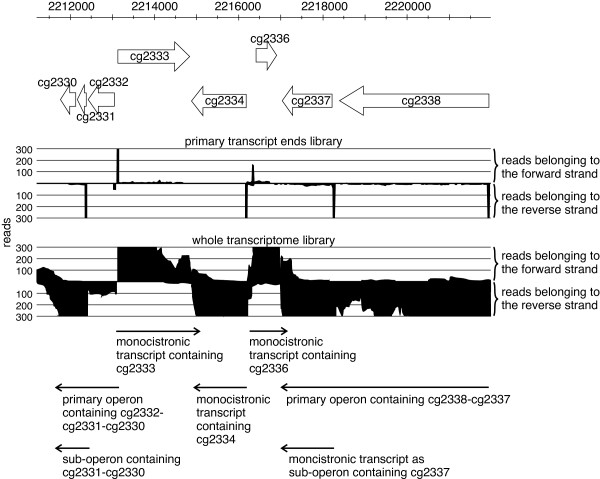
**Classification and identification of operon structures in *C. glutamicum *shown for an example region.** Black color denotes cumulated reads derived from primary transcripts (upper part) or from the whole transcriptome (bottom part) that are both used to detect operon structures. The y- and x-axis represents coverage and genomic position. Primary operons that were found by combined read pairs covering neighboring genes: *cg2332-cg2331-cg2330,* and *cg2338-cg2337*; monocistronic operons that were indicated by reads covering only one gene: *cg2333, cg2334,* and *cg2336*; sub-operons that were identified by TSSs of the primary transcript ends library (stacks in the upper part) within primary operons: *cg2331-cg2330,* and *cg2337.*

Due to the usage of the developed whole transcriptome protocol and the paired-end sequencing, as well as the primary 5′-end data, we were able to identify operon structures in *C. glutamicum* and could assign genes to monocistronic transcripts, primary operons, and sub-operons. Genes were assigned to a primary operon, if 15 or more combined read pairs connect neighboring genes. Thus, a primary operon builds a chain of co-transcribed genes. All remaining genes that could not be assigned to primary operons were categorizes as monocistronic transcripts. As RNAseq data from the whole transcriptome as well as data from the primary 5′-ends were available and can be combined, in several cases we found that polycistronic operons have internal transcription starts and posterior genes might also form alternative sub-operons [15,21] within the larger primary operon (Figure 6).

Altogether, 1,943 annotated genes could be assigned to 616 primary operons, including 565 sub-operon structures (Additional file 1: Table S5). Furthermore, this analysis showed that two-thirds of the estimated ~3,000 genes in *C. glutamicum* are transcribed as operons and one-third monocistronically (1,013 in total).

Additionally, the non-coding RNAs 6C RNA, and 4.5S RNA were identified as monocistronic transcripts, and the M1 RNA was assigned to a primary operon as it was found co-transcribed with annotated protein-coding genes. Furthermore, 8 tRNAs were also found to be co-transcribed with protein-coding genes (Additional file 1: Table S5). The remaining 87 genes that could not be allocated to primary operons or monocistronic transcripts had very low transcript levels (mean coverage below 1).

**Figure 7:**
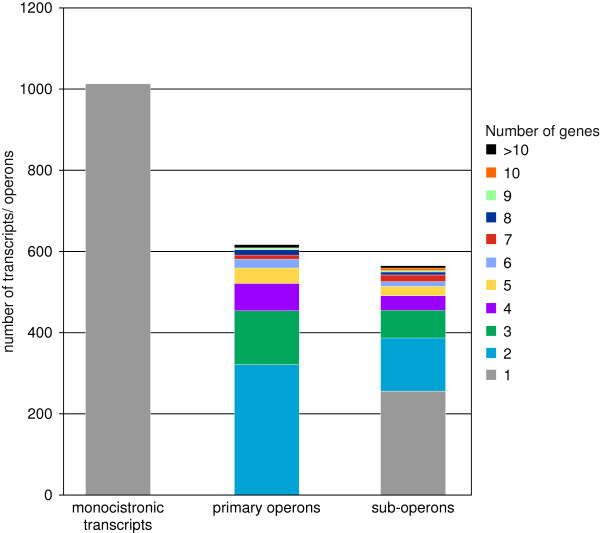
**Analysis of the gene numbers in monocistronic transcripts, primary operons, and sub-operons in *C. glutamicum.*** Operons differing in the number of genes are shown in different colors.

Generally, the quantity of operons decreases with increasing number of genes (Figure 7). Thus, only 9 primary operons contain 10 or more genes (Table 3), whereas 320 primary operons contain 2 genes. The largest primary operon identified here contains 16 genes coding for proteins with diverse cellular functions (translation, ribosomal structure and biogenesis, transcription, replication, recombination and repair, carbohydrate transport and metabolism, and coenzyme transport and metabolism, according to *eggNOG* classification [47]). Upon manual inspection, the gene functions of this large transcription unit comprise riboflavin biosynthesis, protein modifying enzymes and the important *S*-adenosylmethionine (SAM) synthase, responsible for the synthesis of the main methyl group donor of the cell, SAM. It was apparent, that also the proteins of other cofactor biosyntheses are organized in long operons. This was the case for heme, cytochrome, folate and nicotinamide adenine dinucleotide (NAD). Other long operons contain functions in protein turnover (ribosomal proteins, chaperons, and the pupylation/proteasome machinery responsible for protein degradation).

**Table 3:** List of the largest identified primary operons (≥ 10 genes)

**Genes**	**Gene number**	**Strand**	**TSS**^**a**^	***eggNOG*** [47]**classification of genes within primary operon**^**b**^
*cg3011* to *cg3020*	10	-	detected	posttranslational modification, protein turnover, chaperones; unknown function
*cg0593* to *cg0604*	11	+	detected	translation, ribosomal structure and biogenesis (ribosomal proteins)
*cg1683* to *cg1693*	11	-	detected	various ^c^ (protein secretion by Tat pathway, pupylation and proteasome functions)
*cg2578* to *cg2589*	11	-	detected	various (proline and NAD biosynthesis)
*cg0414* to *cg0424*	11	+	detected	signal transduction mechanisms; cell wall/membrane/envelope biogenesis; amino acid transport and metabolism; unknown function (cell envelope, glycan structures)
*cg2974* to *cg2987*	13	-	detected	various (lysyl-tRNA synthase, folate biosynthesis, nucleotide salvage)
*cg0510* to *cg0524*	14	+	detected	carbohydrate transport and metabolism; coenzyme transport and metabolism; posttranslational modification, protein turnover, chaperones; inorganic ion transport and metabolism; unknown function (heme and cytochrome c biosynthesis)
*cg2363* to *cg2377*	15	-	detected	cell cycle control, cell division; cell wall/membrane/envelope biogenesis; unknown function (peptidoglycan biosynthesis, cell division)
*cg1792* to *cg1807*	16	-	detected	various (riboflavin biosynthesis, protein modification, *S*-adenosylmethionine synthesis)

^a^According to the first gene in operon; detected within the primary 5′-transcript ends data.

^b^Functional classes based on manual inspection are included in brackets.

^c^Genes within one primary operon are classified to more than 5 different *eggNOG*[47] functions.

### Description and classification of novel transcripts

Besides the validation of already known genes, the obtained transcriptome data was mined to identify new transcripts that were so far unknown for *C. glutamicum*. For description of the start of a new transcript the primary 5′-end as well as the whole transcriptome data was used. For the determination of the 3′-end of a new transcript the whole transcriptome data was applied. Due to the constraints used for the construction of the cDNA library, the data mostly contain transcripts that are larger than 200 nt. The identification and characterization of small RNAs in *C. glutamicum* using RNAseq was studied in detail separately [9].

Altogether, the newly found transcripts were classified into three categories: intergenic transcripts that are located between annotated genes, antisense transcripts that are located on the opposite strand of a transcripts and transcripts that start within a CDS.

In total, our data revealed 916 novel transcripts for *C. glutamicum*. Of these, 30 were identified as intergenic transcripts, 700 as antisense transcripts and 186 as intragenic transcripts (Additional file 1: Tables S6, S7 and S8).

For 18 of the 30 intergenic transcripts, a TSS was identified from the primary 5′-end data set. As intergenic transcripts might encode proteins, the sequences of these intergenic transcripts were scanned for open reading frames using the web program *ORF finder*[46], resulting in the identification of at least one open reading frame for 29 transcripts. In total, 20 of 29 identified open reading frames had at least one homologous protein in another species. Furthermore, to obtain additional evidence for encoded proteins within the new intergenic transcripts, a RBS was searched upstream of the longest predicted open reading frame. While one transcript was predicted to be leaderless, a potential RBS was identified for 13 of the 28 remaining transcripts (Additional file 1: Table S6).

In total, 700 new transcripts were classified as antisense transcripts. Of these, 696 are located in antisense orientation to one or more annotated protein-coding sequences. The remaining four antisense transcripts are located on the opposite strand of other transcripts (Additional file 1: Table S7). Yet, only for 233 of those antisense transcripts a clear TSS could be identified. The remaining transcripts probably arise from σ^A^-independent transcription events occurring only under stress conditions since they were found in the total transcriptome library but not in the 5′-enriched data set.

### Transcript ends and predicted rho-independent terminators

**Figure 8:**
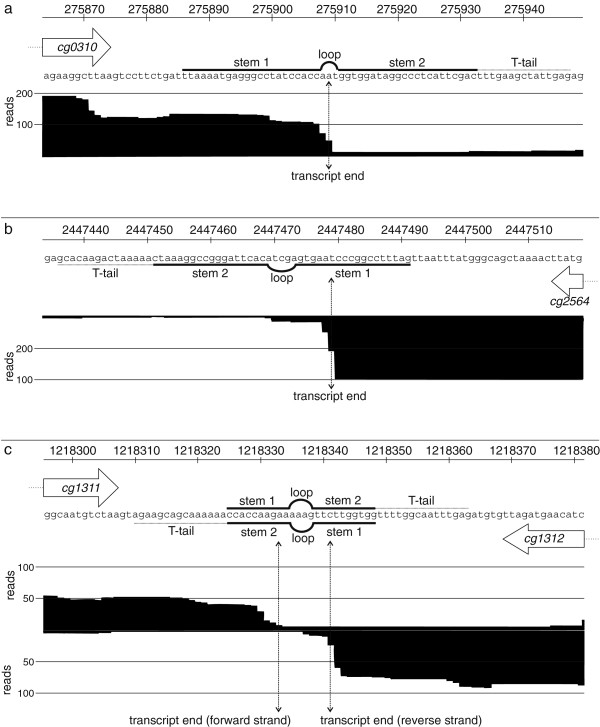
**Examples of transcript ends determined by RNAseq and predicted rho-independent terminators.** The genomic regions, predicted terminators, and accumulated reads were shown. Protein-coding regions are indicated by arrows. The picture includes two examples of unidirectional terminators **(a and b)** and one example of a bidirectional terminator structure **(c)**. Rho-independent terminators were predicted by *TransTermHP*[54].

In addition to the analysis of transcription start sites and operon structures, we also examined the 3′-transcript ends and compared those to rho-independent terminators predicted by *TransTermHP*[54]. Therefore, we extracted the *TransTermHP* predictions for all monocistronic transcripts and the last gene within the defined primary operons. Overall, we obtained 1,383 predicted intrinsic terminators and for only 320 (23.1%) of these a clear transcript end could be determined, based on the following criteria: the mean coverage of the last 5 bases before the first terminator stem must be at least 11 and the ratio of this mean coverage and the coverage of a given base within the terminator must be ≥ 5. Of the remaining 1,063 predicted terminators, 290 (21%) transcript ends were insufficiently covered and 773 (55.9%) displayed only a gradual decrease in coverage. For the 320 predicted rho-independent terminators we found that the majority of transcript ends were located within stem 1 of the predicted rho-independent terminator and the minority within the loop, stem 2, or T-tail (72.2% within stem 1, 18.4% within the loop, 7.5% within stem 2, and 1.9% within the T-tail; Figure 8, Additional file 1: Table S9). It was surprising that only a low number of 3′-ends were located in the T-tail.

## Discussion

This study presents the first comprehensive transcriptome analysis of *Corynebacterium glutamicum*. The complete genome sequence of *C. glutamicum* ATCC 13032 and its annotation were published in 2003 [4,5], but knowledge on transcriptional organization, promoter elements, and non-coding transcripts was known only for a few selected examples.

The use of RNAseq to analyze and characterize the transcriptomes in prokaryotic as well as in eukaryotic cells has been exploited in recent times [15,17,26,55-58]. While commercial RNAseq kits were used in most of the above–mentioned studies, here, we introduce two improved RNAseq library preparation protocols which provide to the possibility to customize each step as required. The described approach can easily be adapted for other species.

For the description of the transcriptome profile of *C. glutamicum*, we combined the results of two RNAseq protocols, one that addresses the primary and the other the whole transcriptome. Combining both data sets, we were able to report defined 5′- and 3′-ends of annotated transcripts, novel transcripts, and operon structures. The sequencing of a library that contained enriched primary transcript ends enabled a mapping of 2,591 TSSs that represented the basis for the analysis of promoter motifs, 5′-UTRs, RBSs and operon structures.

### σ^A^-dependent promoters in *C. glutamicum*

The search for recurring motifs at the 2,591 TSSs revealed a -10 region (TAnnnT) and a less conserved -35 region (ttgnca) of the sigma factor σ^A^-dependent promoter in *C. glutamicum*. This primary sigma factor is present in all known eubacteria [8,37,38] and in *C. glutamicum* its reported recognition sequence consists of two hexamers, TTGNCA and TANANT, located around the positions -35 and -10 relative to the TSS [8] both of which closely match our results. It was previously observed that *C. glutamicum* promoters do not have well conserved -35 regions [8,44]. The weak conservation of the -35 motif in *C. glutamicum* can be explained by the fact that there is a high number of promoters with an extended -10 promoter element, TGnTATAAT [41,59,60], able to specify the full functionality for sigma factor recognition. In addition to the house-keeping σ^A^ and the primary-like σ^B^, *C. glutamicum* possesses five additional sigma factors of the ECF family (σ^C^, σ^D^, σ^E^, σ^H^, and σ^M^), each responsible for recognizing promoters of genes involved in specific functions and stress responses [8]. These sigma factors might be responsible for recognition of promoters under stress conditions that were analyzed only in the whole transcriptome sample. Since the RNA sample for 5′-enriched library was generated from unstressed conditions, promoter analyses were only performed for putative housekeeping promoters. Additional analyses of these promoters and their motifs will be performed in future studies (Albersmeier *et al.*, in preparation).

The identification of TSSs also revealed that 365 genes contain multiple TSSs (up to six) that result in different lengths of the 5′-UTR. The occurrence of multiple TSSs can be explained by the presence of multiple promoters. The use of multiple promoters might be helpful for adaption to nutritional signals, with one promoter ensuring a constitutive gene expression and the others increased transcription in presence of specific stimuli [61].

### Characterization of 5′-UTRs indicate novel regulatory elements

Our analysis confirmed the existence of 13 5′-UTRs that have been predicted by the *Rfam* database [50], among them several riboswitches (e.g. 5 thiamin pyrophosphate-sensing riboswitches as 5′-UTRs of *cg0825, cg1227, cg1476, cg1655, and cg2236*, one *S*-adenosylmethionine-sensing riboswitch as 5′-UTR of *cg1478*, and one flavin mononucleotide-sensing riboswitch as 5′-UTR of *cg0083*). Riboswitches control gene expression at the mRNA level by undergoing conformational changes. A change is usually induced upon binding of small molecules that provide chemical moieties to interact with nucleic acids, often products of metabolic pathways they thereby regulate [48,52,53]. The occurrence of riboswitches in the 5′-UTR region of metabolic genes that directly bind diverse metabolites and influence both at transcriptional and translational level have been described for Gram-positive and Gram-negative bacteria [52,53,62,63]. For *C. glutamicum*, the existence and function of these riboswitches is indeed likely as some genes containing putative riboswitches within their 5′-UTR are annotated as enzymes with a clearly associated metabolic function. In other cases, the existence of a riboswitch of a certain class will be helpful in identification of the functional context of this gene. Beside the metabolite-sensing riboswitches, one RNA thermometer (within the 5′-UTR of *cg0215*) could also be validated by the annotation of its gene as putative cold-shock protein (*cspA*). In *E. coli* the *cspA* thermosensor is described to modulate the translation of the *cspA* mRNA (encoding a cold-shock protein) more efficiently at low temperatures than at higher temperatures, resulting in the adaption of cellular mechanisms at different temperatures as a cold-shock response [64]. The structural prediction of the RNA thermometer within the 5′-UTR of *cg0215* and the cold-shock protein encoded by *cg0215* mRNA indicate a similar function of the putative RNA thermometer in *C. glutamicum,* although this remains to be proven experimentally. Moreover, a wealth of candidates that might harbor novel *cis*-regulatory RNA structures was also identified. For example, genes involved in the metabolism of amino acids (e.g. arginine, cysteine, histidine, and methionine) contain 5′-UTRs longer than 60 nt that might contain riboswitch-like structures (e.g. *cg2305/hisD*[65]). Previously, several RNA elements have been described to regulate amino acid metabolism genes in bacteria [52,53,66]. Such *cis*-regulatory elements allow a fine-tuning of gene expression [67], and this might be advantageous for fast-growing, metabolically versatile bacteria like *C. glutamicum.* Beside the occurrence of riboswitches and RNA thermometers within the 5′-UTRs of mRNAs, stem-loop structures of 5′-UTR which have a protective effect against mRNA degradation play a role in mRNA decay [68,69]. This might also be an explanation for the relatively high number of 5′-UTRs longer than 60 nt in *C. glutamicum*.

### Abundance of leaderless mRNAs in *C. glutamicum*

Approximately 33% of mRNAs were identified to be leaderless in *C. glutamicum.* Previous transcriptional studies have revealed ~1.5% (26 of 1,907) leaderless mRNAs in *Helicobacter pylori*[15], ~28% (53 of 192 with a 5′-UTR < 11 nt) in *Streptomyces coelicolor*[32], and ~4.7% (8 of 170 with a 5′-UTR < 11 nt) in *Pseudomonas putida* KT2440 [26], so that the fraction of leaderless transcripts (707 of 2,147 mRNAs) in *C. glutamicum* is the highest described in any bacterium. It is already known that leaderless transcripts, although occurring in many taxonomic branches of bacteria, are especially abundant in Gram-positives [70]. Interestingly, it was previously found that archaeal genes are commonly expressed as leaderless transcripts [71,72], although there are also archaea which carry mainly long 5′-UTRs [73]. However, it is obscure whether there is a similarity between archaeal transcription and the transcriptional machinery of *C. glutamicum* since the transcription in archaea resembles more closely that in eukaryotes [74].

The translational mechanism of leaderless transcripts and the recognition of such mRNAs without a 5′-UTR and a canonical RBS by the ribosomes were unknown for a long time. However, in *E. coli* it was shown that the AUG start codon and the initiator tRNA can promote stable binding between the mRNA and the ribosome [70,75], and that leaderless transcripts have a preference to 70S ribosomal monomers over 30S subunits, proposing an alternative translation initiation pathway for such mRNAs [76]. The work presented here revealed AUG (~79%) and GUG (~21%) as initiation codon for leaderless transcripts in *C. glutamicum*. Although the mechanism, by which GUG can replace AUG in leaderless translation is not clear, it is described that GUG might function as start codon in leaderless transcripts also in other organisms, potentially providing a lower translational level [32,70]. It can be assumed that leaderless transcripts might be remnants of ancestral mRNAs, because heterologous leaderless mRNAs derived from bacteria can be reliably translated in archaea and eukaryotes [70]. The commonality and simplicity in the translation initiation on leaderless mRNAs in archaea, bacteria, and eukaryotes is also indicative for an early evolutionary origin of such a translation mechanism [77].

### RNAseq data reveal AGGag as ribosome binding site for *C. glutamicum*

Based on our data, it was possible to obtain the most accurate and comprehensive analysis of the RBS in *C. glutamicum* so far. Our data show an increase in the fraction of purines (> 55%) in the region between the 6th and 18th nt upstream of the initiation codon, indicating a recognition region for the ribosome [28]. The search for a motif within this region identified the conserved motif AGGag in about 89% of 5′-UTR sequences in *C. glutamicum* that represents the reverse complement of the 3′-terminus of the 16S rRNA*.* These discoveries fit very well to previously published data for *E. coli*[28,78-80]*.* Beside the initiation codon, the translation efficiency is also dependent on the hybridization of the RBS of the mRNA to the 3′-end of the 16S rRNA, and thus, on its sequence conservation [81,82]. It was shown that translation can be increased using optimal RBS that are perfectly complementary to the 3′-terminus of the 16S rRNA [81,82]. Therefore, it is likely that the most conserved bases from the 1st to the 3rd position (AGG) within the RBS motif AGGag in *C. glutamicum* are essential for the translation initiation mechanism, whereas the remaining bases modulate translation efficiency. The spacing between RBS and start codon was also shown to have a strong effect on translation, suggesting that the physical distance between the 3′-terminus of the 16S rRNA and the anti-codon of the initiator fMet-tRNA [79,83] is of importance. In *C. glutamicum*, a spacing between 4 - 12 nt was found to be the most common (> 90%). The calculated mean spacing of 7.7 ± 2.7 nt fits well to the number derived for *E. coli* (mean spacing of 6.9 ± 2 nt) [84]. Therefore, approx. 5 - 10 nt is likely to be the “optimal” spacing.

### Revealing operon structures for *C. glutamicum*

In this RNAseq approach, about 97% of the annotated genes were found to be transcribed under at least one of the nine different growth conditions used. Besides validating the annotated coding sequence, the genes could furthermore be assigned to 616 primary operons or to 1,013 monocistronic transcripts. Genes that are located next to each other and are transcribed coordinately from a single promoter form a genetic unit, an operon [27,85]. Due to the resolving power of RNAseq, executed on 5′-ends of native transcripts, it was furthermore found that 565 sub-operons are present. The existence of additional sub-operons was previously described for different bacteria: for example, the mapping of TSSs in *Helicobacter pylori* identified 337 primary operons, 126 sub-operons, and 66 monocistrons within the primary operons [15], whereas in *Mycoplasma pneumoniae* 341 identified operons could be divided into 447 smaller alternative transcription units by analyzing transcription at 173 different conditions [21]. The use of internal TSSs greatly increases the transcriptomic complexity and regulatory capacity [22,86]. Furthermore, other transcriptomic analyses revealed a condition-dependent modulation of operon structures. In such cases, a gene assigned to a polycistron in one condition can be transcribed as a monocistron in another condition [21,87] suggesting switchable operon structures [22].

In addition to the primary operons, we found that a third of the protein-coding regions in *C. glutamicum* are located on transcripts that are transcribed monocistronic. This large number indicates that in general, the advantage to regulate each gene individually, e.g. by transcription factors, outweighs the advantage of coordinated expression as part of a polycistron as well as the drawback of an increasing “regulatory burden” associated with an increasing number of monocistrons [67].

On the other hand, we found that genes involved in the synthesis of enzyme cofactors or in protein metabolism (from translation to degradation in the proteasome machinery) are organized in long operons. This finding might be interpreted that especially in these cases coordinated expression is the major organizational force.

### Detection of novel transcripts unveil a huge amount of antisense transcription

Our data revealed 916 transcriptionally active, but yet unknown regions for *C. glutamicum*. Out of them, 30 were identified as intergenic, 186 as intragenic, and 700 as antisense transcripts (Additional file 1: Tables S6, S7 and S8). The intergenic transcripts most probably represent genes by themselves, either encoding small proteins or being a non-coding RNA. Small, non-coding RNAs are widely distributed in bacteria and also present in high numbers in *C. glutamicum*. Since the small RNA fraction of the bacterial transcriptome requires special preparation protocols for analysis by RNAseq, it is not properly represented in the data sets obtained here and subject of another study that addresses the small RNA fraction of the *C. glutamicum* transcriptome [9].

Quite a high number of intragenic TSSs (186 in total) that mapped within an annotated gene were determined for *C. glutamicum*. Yet, it is not clear whether this TSSs result in alternative, shorter proteins, novel protein-coding or non-coding genes. Such internal TSSs were also described for *H. pylori*[15]. Furthermore, such internal TSSs were also found in viruses and in human, producing a second, shorter gene product [88-90]. It is speculated that such internal transcripts that might harbor multi-functional, possibly regulatory regions increase the genomic information content [90].

Furthermore, 700 new antisense transcripts were identified for *C. glutamicum*. Yet, only for 233 of those a TSS was mapped. It is likely that the remaining transcripts arise from sigma factor σ^A^-independent transcription present only under stress conditions since they appeared in the library that included RNA from stress conditions but not in the other that was built from normal growth conditions. These antisense transcripts are expected to be non-coding RNAs and may have regulatory roles in gene expression. Strand-specific transcriptome sequencing has led to the description of massive amounts of antisense RNAs in various bacteria, eukaryotes, and archaea, and might represent a common form of regulation within all domains of life [22,91].

Antisense RNAs act on gene expression by a variety of different mechanisms. On one hand, they can hybridize to a part or the whole sense transcript, causing structural changes in the target affecting transcription termination (attenuation), introduce or block cleavage sites for ribonucleases (RNA cleavage), or have an effect on translation of the target gene by blocking or releasing the RBS (translation block) [91]. On the other hand, their transcription can alter sense transcription by interference, suppressing the production of the transcript due to polymerase collisions (transcription interference) [91]. The use of antisense RNAs for controlling gene expression allows an additional and tight regulation of target genes [91], and permits adaptation of gene expression to more different conditions. For *C. glutamicum*, the function and the regulation of expression of these antisense RNAs is hitherto unknown, but represent an interesting research target for future studies.

### Transcript ends and predicted rho-independent terminators

Rho-dependent and -independent termination are principal mechanisms by which bacterial transcription units are defined [92]. Intrinsic, rho-independent terminators are composed of a hairpin structure with G + C-rich stem followed by a T-stretch [93]. It is proposed that the RNA polymerase complex is destabilized after transcription and formation of the hairpin structure and that the transcribed RNA molecule is released within the T-stretch region [92,94].

The RNAseq data obtained here shows that transcription termination in *C. glutamicum* seems to take place within the T-tail region in only a minor number of transcripts. The most 3′-ends of transcripts were found in the first G + C-rich region of the intrinsic terminator. This observation is contradictory to the literature [92,94]. A possible explanation might be an RNA processing within the stem structure of the terminator by RNases that cleave double-stranded RNA molecules. For *E. coli* it was shown that a read-through of the *int* gene (encodes a protein involved in site-specific recombination) leads to a formation of an RNase III cleavage site within the terminator to regulate the expression of this gene [95]. Since the chosen criteria were a sharp drop-off in the number of reads within the terminator, we might have selected for transcripts in which processing of double stranded RNA occurred. Another explanation for the transcript ends within the terminator stem might be that a cruciform structure formed on the DNA within the terminator region displaces the RNA polymerase thereby terminating transcription in the region of the terminator stem. Although it was shown *in vitro* that A + T-rich sequences can more easily form cruciform structures than G + C-rich sequences, it was never shown *in vivo*[96].

However, we cannot exclude that the use of the stem-loop DNA adapter with seven wobble bases did not hybridize well in such stem-loop regions and therefore did not reach a perfect resolution of 3′-transcript ends. To prove or disprove this and to get a better resolution of the 3′-transcript ends, other library preparation methods might be used. Suitable methods are already described for eukaryotic polyA-RNA: RNA-PET (RNA-paired end tagging). In RNA-PET the 5′- and 3′-end tags of full-length cDNA fragments are fused and subsequently sequenced [97].

It should be mentioned that for about 80% of the predicted rho-independent terminators for *C. glutamicum* no clear transcript end could be determined, either because of insufficient sequence coverage or because of a gradual and slow transcription drop-off. This indicates that the connection between RNAseq analysis and 3′-ends or the transcripts or the processes involved in rho-independent termination in *C. glutamicum* or more general in actinobacteria are not yet fully understood.

## Conclusions

In this study we have created the *C. glutamicum* transcriptome profiles using an improved RNAseq method. The generated transcriptome data reported in this manuscript is strand-specific and allows novel insights into the transcriptomic organization and a comprehensive discovery of novel transcripts from intergenic and antisense regions throughout the genome. We were able to map and describe operon structures for this bacterium and classified more than 70% of the new transcripts as antisense transcripts. We identified more than 2,000 TSSs supported by promoter search of the housekeeping sigma factor σ^A^ and re-annotated more than 200 gene starts. Surprisingly, we observed that about 33% of the mRNAs in *C. glutamicum* are leaderless transcripts without any preference for functional categories of the proteins encoded by these transcripts. In this sense, it appears to be the ultimate tool for an information-rich genome annotation. The transcriptomic data established in this study deliver an enormous amount of information on various subjects and opens many new fields to be further investigated. Among these are hitherto unknown transcriptional mechanisms in *C. glutamicum* and transcription-based regulation on the transcriptional as well as on the translational level.

## Methods

### Bacterial strains, oligonucleotide and culture media

The bacterial strain used in this study is *Corynebacterium glutamicum* ATCC 13032. The oligonucleotides used are listed in Table 4. For shaking flask cultivation, *C. glutamicum* was grown in the complex medium LB (lysogeny broth) or in the chemically defined medium CGXII [98], but containing only 2% glucose (instead of 4%) and 30 mg l^-1^ instead of 0.03 mg l^-1^ protocatechuic acid, in 250 ml shaking flasks at 30°C and 300 rpm. The fermenter minimal medium was a derivate of CGXII medium containing 25 g l^-1^ NH_4_(SO_4_)_2_, 1 g l^-1^ KH_2_PO_4_, 0.25 g l^-1^ MgSO_4_, 10 mg l^-1^ FeSO_4_ · 7H_2_O, 10 mg l^-1^ MnSO_4_ · H_2_O, 1 mg l^-1^ ZnSO_4_ · 7H_2_O, 0.2 mg l^-1^ CuSO_4_ · 5H_2_O, 0.02 mg l^-1^ NiCl_2_ · 6H_2_O, 10 mg l^-1^ CaCl_2_ · 2H_2_O, 2 mg l^-1^ biotin, 30 mg l^-1^ protocatechuic acid, and 4% glucose. For cultivation of *C. glutamicum* in a fermenter system, pre-cultures were grown in CGXII minimal medium in shaking flasks. The fermenter was then inoculated with pre-culture corresponding to a start OD_600 nm_ (optical density) of 0.2. The fermentations were performed in 1 l Biostat Q bioreactors (Sartorius, Göttingen, Germany) at pH 7, 30°C and 30% dissolved oxygen.

**Table 4:** Oligonucleotides used as adapters and primers in this study

**Name**	**Sequence (5′-3’)**
RNA adapter	CCCUACACGACGCUCUUCCGAUCGAG
stem-loop DNA adapter	AGATCGGAAGAGAGACGTGTGCTCTTCCGATCTNNNNNNN
amplification primer 1	AATGATACGGCGACCACCGAGATCTACACTCTTTCCCTACACGACGCTCTTCCGATCGAG
amplification primer 2	CAAGCAGAAGACGGCATACGAGATCGTGATGTGACTGGAGTTCAGACGTGTGCTCTTCCGATCT

All oligonucleotides used in this work were synthesized at Metabion International AG (Martinsried, Germany).

### Growth conditions for cell sampling

To obtain the RNA for enrichment of primary transcripts as well as for the whole transcriptome, *C. glutamicum* cells were grown in shaking flasks in complex LB medium and defined CGXII medium as described above and harvested in the exponential growth phase.

For the whole transcriptome sample, *C. glutamicum* cells were additionally grown in shaking flasks in defined CGXII medium and subjected to different stresses at an OD_600 nm_ of 10. High salt stress: addition of sodium chloride to a final concentration of 10%. Heat shock stress: shift of the temperature from 30°C to 50°C. Cold shock stress: temperature shift from 30°C to 4°C. Alcohol stress: addition of ethanol to a final concentration of 10%. Oxidative stress: addition of diamide to a final concentration of 2 mM; addition of hydrogen peroxide to a final concentration of 1%. In each case, cells were harvested 15 min after exposure to stress conditions. Additionally, *C. glutamicum* cells were grown in a fermenter in defined CGXII medium to be able to apply high oxygen stress. Therefore, the dissolved oxygen level was shifted from 30% to 90%. After 2 hours of 90% dissolved oxygen cells were harvested.

### RNA isolation procedures

For construction of the primary 5′-end library, total RNA was isolated using the RNeasy mini kit along with an RNase-free DNase set (Qiagen, Hilden, Germany) and a DNase I kit (Roche Diagnostics, Mannheim, Germany) according to Hüser *et al.* (2003) [99].

For creation of the whole transcriptome library, total RNA was isolated using TRIzol (Life Technologies, Darmstadt, Germany). The cell pellet obtained from 800 μl of exponentially grown culture was resuspended in 1 ml TRIzol reagent. Cell disruption and homogenization was performed using the homogenizer Precellys 24 (Bertin Technologies, Montigny-le-Bretonneux, France) at a speed of 6.5 for 20 sec twice. After centrifugation at 16,000 g for 3 min at 4°C, 200 μl chloroform (Roth, Karlsruhe, Germany) was added to the supernatant and shaken vigorously for 30 sec followed by incubation for 1 min at room temperature and centrifugation at 16,000 g for 10 min at 4°C. For precipitation, 1 volume of isopropanol (Roth, Karlsruhe, Germany) was added to the aqueous supernatant and shaken vigorously. The sample was then incubated on ice for 10 min and centrifuged at 16,000 × g (times gravity) for 15 min at 4°C. The RNA pellet was washed two times with 75% (v/v) ethanol, air-dried and dissolved in 100 μl deionized, RNase-free water. The total RNA isolation using TRIzol reagent was followed by DNase treatment with an RNase-Free DNase Set (Qiagen, Hilden, Germany) and a DNase I kit (Sigma-Aldrich, Hamburg, Germany) according to manufacturer’s instructions.

To ensure DNA-free RNA samples, a PCR was performed using oligonucleotides which create two different products of about 150 bp and 500 bp. Afterwards, the purified total RNA was quantified with a NanoDrop 1000 spectrometer (Peqlab, Erlangen, Germany) and qualified by Agilent RNA Nano 6000 kit on Agilent 2100 Bioanalyzer (Agilent Technologies, Böblingen, Germany).

### The native 5′-end protocol

To analyze the native transcription start sites of *C. glutamicum* a native 5′-end RNAseq protocol was developed. The protocol starts with 10 μg column-based isolated total RNA. An essential step for effective transcriptome sequencing is the subsequent depletion of stable RNA (rRNA, tRNA) that can constitute more than 95% of a bacterial transcriptome. Here, we used the hybridization-based Ribo-Zero rRNA removal kit for Gram-positive bacteria that showed an advantageous depletion in the amount of stable RNA for *C. glutamicum*. Then, the depleted RNA was fragmented to a size of 200 - 500 nt. Primary transcript ends were enriched using Terminator 5′-phosphate-dependent exonuclease (Epicentre, Madison, WI, U.S.A.). This enzyme recognizes and digests processed, non-primary transcripts that offer a monophosphate at their 5′-end. Bacterial primary transcripts that possess three phosphates at their 5′-ends are not digested and remain in the solution. To be able to ligate RNA adapters at the 5′-ends, the resulting 5′-triphosphate ends were processed to 5′-monophosphate ends by RNA 5′-polyphosphatase (Epicentre, Madison, WI, U.S.A.). The tagging of the 3′-end of the RNA fragments and the reverse transcription into cDNA is performed ligation-free with a stem-loop DNA adapter with seven free wobble bases at its 3′-end. The wobble bases hybridize to the 3′-end of a RNA fragment serving as a primer for the reverse transcriptase. The advantage of the use of a stem-loop DNA adapter lies in the avoidance of ligation of an adapter at the 3′-end which can be inefficient depending on the adapter sequence [100] and length of the RNA [101,102]. Additionally, it was shown previously that the use of stem-loop primers for reverse transcription is better than conventional primers in terms of efficiency and specificity [103]. After reverse transcription of the RNA fragments into cDNA, the cDNA fragments were amplified and are then ready for sequencing. The procedure of the native 5′-end protocol is depicted in Figure 1b.

The detailed steps of the library preparation are described below.

### The whole transcriptome protocol

As a completion to the native 5′-end protocol a whole transcriptome protocol was also developed that enables the sequencing of all transcripts. The procedure of this protocol is very similar to the procedure of the native 5′-end protocol. The difference between both protocols is the missing enrichment step for primary transcripts and an additional step before the RNA adapter is ligated, where unphosphorylated 5′-ends were phosphorylated and phosphorylated 3′-ends were dephosphorylated using a T4 polynucleotide kinase (New England BioLabs, Frankfurt am Main, Germany). The procedure of the whole transcriptome protocol is shown in Figure 1a.

### Depletion of ribosomal RNA

For depletion of ribosomal RNA, the Ribo-Zero rRNA removal kit Gram-positive bacteria (Epicentre, Madison, WI, U.S.A.) and purification of the rRNA-depleted sample by ethanol precipitation was used according to manufacturer’s instructions.

The purified RNA was then pooled in equimolar amounts (for primary 5′-end library derived from two cultivation conditions and for whole transcriptome library derived from nine cultivation conditions as described above) and in total 10 μg RNA were taken for depletion of rRNA.

### Phenol-Chloroform Isoamyl alcohol extraction

After enzyme treatments, the RNA was purified by phenol-chloroform isoamyl alcohol, 25:24:1, (PCI) extraction. One volume of PCI was added to the sample and shaken vigorously for 30 sec followed by centrifugation at 16,000 × g for 15 min at room temperature. For precipitation, 0.3 volumes of sodium acetate (3 M; pH 5.2), 20 μg of glycogen (RNA grade; Thermo Fisher Scientific, Schwerte, Germany) and 2.7 volumes of ice-cold ethanol (~99%) were added to the aqueous supernatant and shaken vigorously. The sample was then incubated at -20°C for at least 2 hours and centrifuged at 16,000 × g for 20 min at 4°C. The RNA pellet was washed two times with 75% (v/v) ethanol, air-dried and dissolved in deionized, RNase-free water, the volume depending on the following reaction step.

### RNA fragmentation

For both protocols, the RNA needs to be fragmented to sizes of 200 - 500 nt. Therefore, 0.25 volumes of fragmentation buffer (100 mM potassium acetate and 30 mM magnesium acetate dissolved in 200 mM Tris–HCl pH 8.1) were added to the RNA sample, shaken vigorously, and incubated at 94°C for 2.5 min. After incubation, the sample was mixed vigorously with one volume of ice-cold fragmentation stop buffer (10 mM Tris, 1 mM EDTA, pH 8) and incubated on ice for 5 min.

After fragmentation, the RNA sample was precipitated with 0.3 volumes of sodium acetate (3 M; pH 5.2), 20 μg of glycogen (RNA grade), and 2.7 volumes of ice-cold ethanol (~99%) at -20°C for at least 2 hours and centrifuged at 16,000 × g for 20 min at 4°C. The RNA pellet was washed two times with 75% (v/v) ethanol, air-dried and dissolved in 40 μl of deionized, RNase-free water.

Next, RNA fragments larger than 150 nt are precipitated with 10 μl of enrichment solution (25% PEG 8000 (polyethylene glycol), 2.5 M sodium acetate in RNase-free water) on ice for 30 min and centrifuged at 16,000 × g for 20 min at 4°C. The RNA pellet was washed two times with 75% (v/v) ethanol, air-dried and dissolved in deionized, RNase-free water, the volume depending on the following reaction step.

### Terminator 5′-phosphate-dependent exonuclease treatment

For the primary 5′-end protocol, a Terminator 5′-phosphate-dependent exonuclease (Epicentre, Madison, WI, U.S.A.) treatment was included to digest the non-primary transcripts. This enzyme treatment was used according to manufacturer′s instructions followed by PCI extraction.

### 5′-end repair

After fragmentation, it is necessary to repair the 5′-RNA ends before ligating adapters. Therefore, the RNA 5′-polyphosphatase (Epicentre, Madison, WI, U.S.A.) was used according to manufacturer’s instructions. This enzyme converts 5′-triphosphorylated and 5′-diphosphorylated RNA to 5′-monophosphorylated RNA. After RNA 5′-polyphosphatase treatment the RNA was purified by PCI extraction.

In the case of the whole transcriptome library, the RNA 5′-polyphosphatase treatment was followed by a T4 polynucleotide kinase treatment (New England BioLabs, Frankfurt am Main, Germany) according to manufacturer’s instructions to phosphorylate unphosphorylated 5′-RNA ends. Instead of the T4 polynucleotide kinase reaction buffer, T4 RNA ligase reaction buffer supplemented with 1 mM ATP was used. After incubation and heat inactivation of T4 polynucleotide kinase the samples are directly used for RNA adapter ligation.

### RNA adapter ligation

An RNA adapter was ligated to the 5′-RNA ends of the prepared RNA fragments. For the ligation, 100 μM of RNA adapter was used with T4 RNA ligase 1 (New England BioLabs, Frankfurt am Main, Germany) according to manufacturer’s instructions.

### Preparation of the stem-loop DNA adapter

Before use of the stem-loop DNA adapter in the reverse transcription reaction, the stem-loop structure has to be prepared. Therefore, a 20 μl aliquot of stem-loop DNA adapter (100 μM) was incubated at 98°C for 3 min and cooled to 25°C at a rate of 1°C per 10 sec in a Mastercycler pro S (Eppendorf).

### Reverse transcription and tagging of the 3′-end of the cDNA in a single step

After ligation of the 5′-adapter, the RNA fragments are reverse transcribed to cDNA using the ThermoScript RT-PCR system (Life Technologies, Darmstadt, Germany) according to manufacturer’s instructions. As cDNA synthesis primer, 1 μl of the prepared stem-loop DNA adapter (100 μM) was used for one library. After adding all ingredients the reverse transcription was performed at 16°C for 30 min followed by 50°C for 1 hour. After heat inactivation of the reverse transcriptase the sample was used for amplification, without removing the RNA template.

### Amplification of the cDNA and purification of cDNA library

The cDNA was amplified with 18 cycles of PCR. For amplification, phusion high-fidelity DNA polymerase (New England BioLabs, Frankfurt am Main, Germany) with 1.5% DMSO was used according to manufacturer’s instructions. The primer annealing was performed at 60°C for 30 sec and the extension at 72°C for 15 sec. After amplification of the cDNA libraries, samples were purified and size-selected (> 150 bp) by using the Agencourt AMPure XP system (Beckman Coulter, Krefeld, Germany) according manufacturer’s instructions. Afterwards, the purified libraries were quantified and qualified with an Agilent 2100 Bioanalyzer (Agilent Technologies, Böblingen, Germany) using the Agilent High Sensitivity DNA kit.

### Sequencing of the cDNA library

Sequencing of the cDNA libraries was carried out on the Genome Analyzer *II*x using TruSeq kits (Illumina, San Diego, CA, U.S.A.). The sample derived from the 5′-end library was sequenced in single read mode with a read length of 27 bases and the whole transcriptome RNAseq library was paired-end sequenced with a read length of 2 × 27 bases. Each sample was sequenced on one lane of a flow cell. Data analysis and base calling were accomplished with the Illumina instrument software. After trimming of one low quality base from the 3'-end of each sequenced read, 26 nt reads were used for further analysis.

The sequencing raw data for both libraries is available from ENA (http://www.ebi.ac.uk/ena/), project ID PRJEB4788.

### Bioinformatics data analysis

#### Read mapping and data visualization

Trimmed reads (26 nt) were mapped to the *C. glutamicum* ATCC 13032 genome sequence [4] with *SARUMAN*[104], allowing for up to one error per read. In case of the mapping of reads belonging to the whole transcriptome library, the forward and reverse read, if both present and with a maximum distance of 1 kb, were combined to one read, containing the reference sequence as insert. Paired mappings with a distance > 1 kb were discarded, and paired reads with either only the forward or only the reverse read mapping were retained as single mapping reads. For the visualization of short read alignments, *ReadXplorer* (Hilker *et al.*, in preparation) was used.

#### Genome-update of *C. glutamicum* ATCC 13032

Since the publication of the *C. glutamicum* genome [4,5] in 2003, knowledge on many gene functions was collected in more than 600 publications. This data was used to update a *GenDB* database [34] that basically contains re-annotated gene starts, some newly annotated genes taken from recent publications and improved gene prediction, and an improved annotation. Furthermore, four non-coding RNAs, 4.5S RNA, 6C RNA, tmRNA, and M1 RNA, which were identified at positions very similar to the Rfam predictions [50] (Additional file 1: Table S10) were also used to update the *C. glutamicum* genome. For the analysis of the RNAseq data, especially for the determination of the transcription start sites and 5′-UTRs, this updated genome was used.

#### Identification of transcription start sites using primary 5′-end data

To automatically and systematically detect TSSs, the mapping data of the library enriched for native 5′-ends was analyzed. First, for each strand and position of the genome, all mappings starting at the given position were counted. As possible TSS all positions on a strand were taken into account that satisfied the following criteria: for a position *i*, the number of read starts *x*_*i*_ on that strand at this position exceeded a background threshold *T* and the ratio *x*_*i*_/*x*_*i*-1_ at this position had to exceed a threshold *R*. After manual inspection of TSSs, *T* was set to 14 and *R* to 5 as these parameters were found to result in a good signal to noise ratio.

Additionally, these automatically detected TSSs, especially those with a distance larger than 300 nt between TSS and TLS, were checked for false-positives based on manual review. A TSS was assumed to be false-positive if no clear accumulation of read starts is observed at the particular position and the found TSS is detected within an uneven gradient of accumulated reads. This specifically applied to TSSs detected within a coding region and/or with a relatively high amount of accumulated reads (> 100), where the above-mentioned parameters are not effective.

#### Identification of novel transcripts

To identify so far unknown transcripts, the data from the whole transcriptome was used. First, for each strand and each position, the coverage was calculated by adding up all mappings at this position. Then, for each strand and each position *i* it is checked whether the coverage *c*_*i*_ crosses the background threshold *T*. For each position that satisfies this criterion it is checked whether the possible transcript can be extended downstream, with each subsequent position *x* satisfying the condition *c*_*i*+*x*_ > *T*. Once a position *x* with *c*_*i*+*x*_ ≤ *T* is reached, this position is considered to be the end of a novel transcript if the transcript does not overlap a known transcript in sense direction.

Every new detected transcript was checked for its genomic context, i.e. whether it is an antisense transcript located antisense to another transcript, an intergenic transcript in between other transcripts, or an intragenic transcript, whereas the transcript start is mapped within an annotated gene. The end position of automatically detected antisense transcripts with length > 1 kb was manually determined using the whole transcriptome data. Furthermore, new transcripts that were not automatically detected, but derived from identified TSSs belonging to antisense or intergenic transcripts were also categorized as new regions. The end positions of those transcripts were also manually identified. The ends of intragenic transcripts could not be identified, because the appropriate transcript overlaps with the transcript of the annotated gene.

Sequences of intergenic regions were searched for open reading frames using the web program *ORF finder*[46] with ATG, GTG, TTG, CTG as start codons, and TAA, TGA, TAG as stop codons. Those sequences where an open reading frame was found were searched for homologous proteins in the NCBI reference proteins database (refseq_protein) for Bacteria and Archaea using the NCBI web services and Blastx (November 2013).

#### Identification of operon structures

For the identification of operons, again the data from the whole transcriptome library was used. For each gene it was tested whether it is connected to a possible neighboring gene downstream in sense direction by at least *T* paired read mappings. All consecutive sets of pairs for which a TSS could be identified for the first gene were considered to constitute a primary operon. Sub operons were then defined by TSSs within the primary operon.

#### Identification of promoter motifs and ribosome binding sites

The identification of RBSs was started with computing the frequency of purines (G and A) compared to that of pyrimidines (T and C) of each nucleotide within identified 5′-UTRs of protein-coding genes. For genes with more than one 5′-UTR, only one 5’-UTR (unique 5′-UTR), but with a minimum length of 20 bases, was used for this analysis. Additionally, all analyzed sequences were trimmed to length of 20 bases. Next, a RBS motif was searched within unique 5′-UTRs with a minimum length of 14 and a trimmed maximum length of 20 bases.

For the identification of promoter and RBSs motifs the web based software tool *Improbizer*[43] was used. Default settings were applied for both searches.

*Improbizer* has identified an extended -10 region (four unpreserved leading bases, a core hexamer, and two or three unpreserved lagging bases, respectively). However, for simplification the identified motif was truncated to the core -10 hexamer and used in this work.

Additionally, identified motifs were described in their conservation by upper or lower case bases. An upper case base is used if it occurs in ≥ 80% of all cases at a certain position within the motif. A lower case base is used, if it arises in > 40% but < 80% of all cases at a certain position within the motif. If a base occurs in ≤ 40% of all cases at a certain position within the motif, a lower case n is used.

## Abbreviations

RNAseq: High-throughput RNA sequencing; TSS(s): Transcription start site(s); TLS: Translational start site(s); UTR: Untranslated region; (q)PCR: Quantitative polymerase chain reaction; RACE: Rapid amplification of cDNA ends; nt: Nucleotide(s); RBS(s): Ribosome binding site(s); bp: Base pair(s); w/o: Without; CPR: Combined pair of reads; FMN: Flavin mononucleotide; TPP: Thiamine pyrophosphate; SAM: *S*-adenosylmethionine; NAD: Nicotinamide adenine dinucleotide; ncRNA(s): Non-coding RNA(s); OD: Optical density; ×g: Times gravity; PCI: Phenol-chloroform isoamyl alcohol; PEG: Polyethylene glycol.

## Competing interests

The authors declare that they have no competing interests.

## Authors’ contributions

KPS carried out the substantial development of the RNAseq protocols, the experimental and data analysis part of the study, and drafted the manuscript. AM helped in the development of the RNAseq protocols. CR participated in the design of the study, performed sequencing and the bioinformatical analysis of raw RNAseq data, and enabled an (semi)-automated analysis of the data. JK coordinated the study and finalized the manuscript. All authors read and approved the manuscript.

## Supplementary Material

Additional file 1: Table S1 List of identified TSSs with −10 and −35 promoter motifs for the primary sigma factor A predicted by Improbizer within 60 bases upstream of the TSSs. **Table S2.** List of genes with corrected genes starts by RNAseq data. **Table S3.** List of protein-coding genes including length and sequence of 5′-UTRs. **Table S4.** List of ribosome binding site motifs within max. 20 nt upstream of the initiation codon in 5′-UTRs. **Table S5.** List of operons, sub-operons, and monocistronic transcripts. **Table S6.** List of yet undescribed intergenic transcripts. **Table S7.** List of yet undescribed antisense transcripts. **Table S8.** List of yet undescribed intragenic transcripts. **Table S9.** Comparison of predicted rho-independent terminator for *C. glutamicum* and the determined transcript ends. **Table S10.** Verification of four ncRNAs predicted by *Rfam* database for *C. glutamicum*. Click here for file

Additional file 2: Figure S1 TSS annotation on the example of the gene *cmt1* (*cg0413*). Black color denotes cumulated reads derived from the primary transcript ends library. The y- and x-axis represent coverage and genome position, respectively. The increase of reads starts is determined at six positions (numbers 1 - 6). Click here for file

Additional file 3: Figure S2 Examples of secondary structures for *Rfam* predicted 5′-UTRs in *C. glutamicum.* Structures were predicted using minimum free energy and the partition function in *RNAfold* provided by the *Vienna RNA web server*[51]. The initiation codon is highlighted in green, the possible RBS in red. Click here for file
