# Supplementary figures and images for: Comprehensive analysis of the Corynebacterium glutamicum transcriptome using an improved RNAseq technique
Source: BMC Genomics. 2013 Dec 17;14:888. doi: 10.1186/1471-2164-14-888 (PMC3890552; doi:10.1186/1471-2164-14-888)

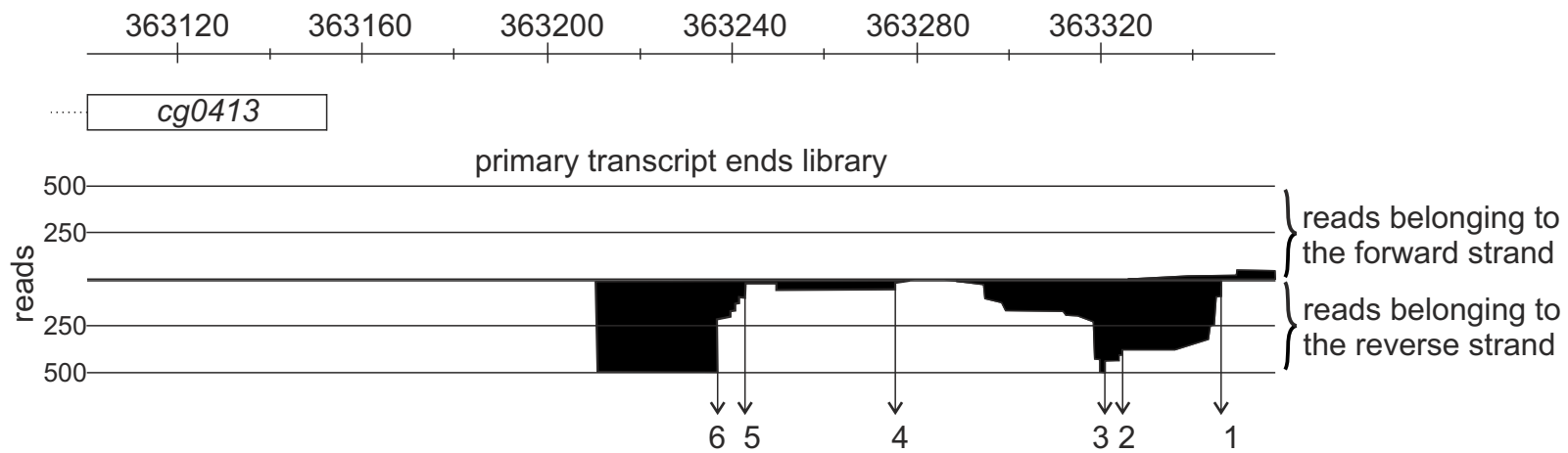

Supplement: Additional file 2: Figure S1 — TSS annotation on the example of the gene cmt1 (cg0413). Black color denotes cumulated reads derived from the primary transcript ends library. The y- and x-axis represent coverage and genome position, respectively. The increase of reads starts is determined at six positions (numbers 1 - 6). [file 1471-2164-14-888-S2.pdf]
