# Supplementary material for: Comprehensive analysis of the Corynebacterium glutamicum transcriptome using an improved RNAseq technique
Source: BMC Genomics. 2013 Dec 17;14:888. doi: 10.1186/1471-2164-14-888 (PMC3890552; doi:10.1186/1471-2164-14-888)

predicted FMN riboswitch - RF 00050
5'-UTR of *cg0083*

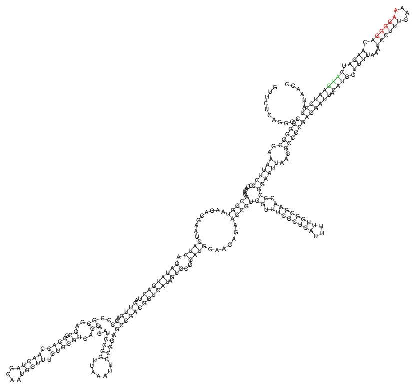

predicted ydaO-yuaA leader - RF 000379
5'-UTR of *cg0936/ rpf1*

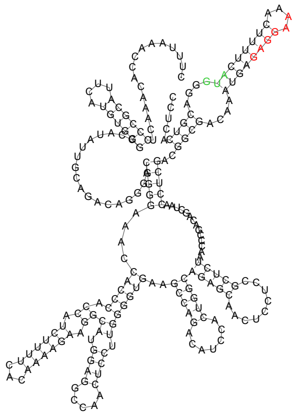

predicted TPP riboswitch - RF 00059
5'-UTR of *cg2236/ thiE*

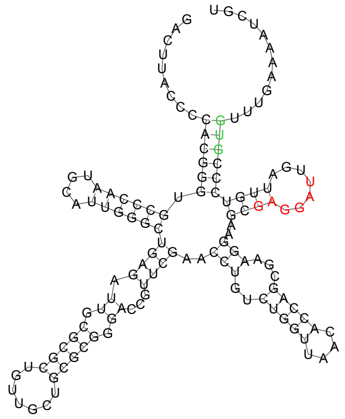

predicted *cspA* mRNA 5'-UTR;
RNA thermometer - RF 01766
5'-UTR of *cg0215/ cspA*

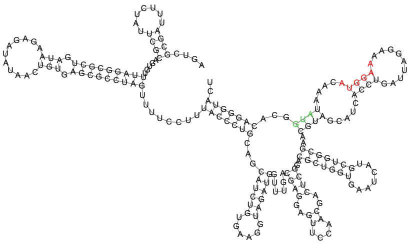

predicted TPP riboswitch - RF 00059
5'-UTR of *cg1227*

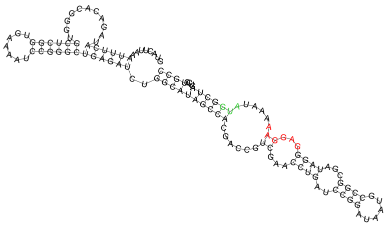

predicted *mraW* RNA motif - RF 01746
5'-UTR of *cg2377/ mraW*

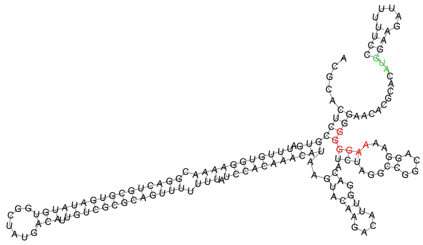

predicted TPP riboswitch - RF 00059
5'-UTR of *cg0825*

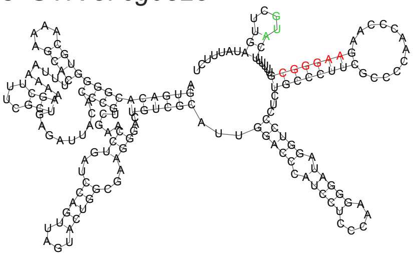

predicted TPP riboswitch - RF 00059
5'-UTR of *cg1476/ thiC*

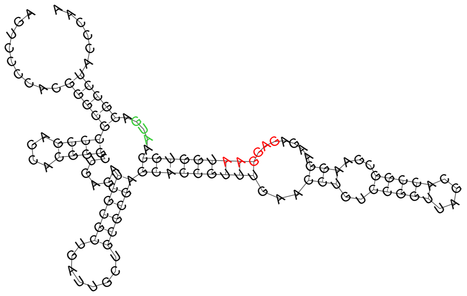

predicted msiK RNA motif - RF 01747
5'-UTR of *cg2708/ msiK*

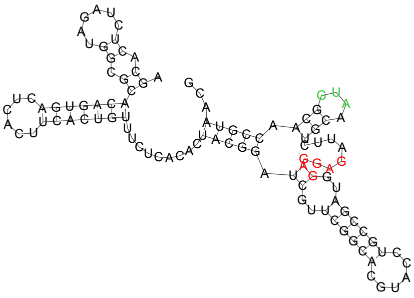

predicted SAM-IV riboswitch - RF 000634
5'-UTR of *cg1478*

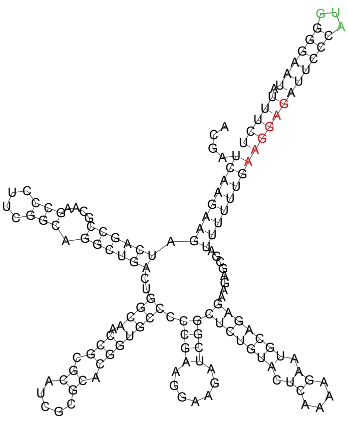

predicted TPP riboswitch - RF 00059
5'-UTR of *cg1655/ thiM*

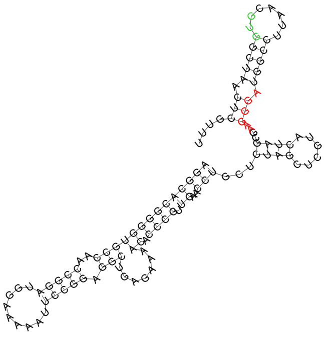

predicted yybP-ykoY leader - RF 00080
5'-UTR of *cg2157/ terC*

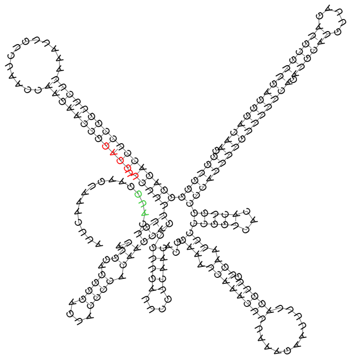

predicted ydaO-yuaA leader - RF 000379
5'-UTR of *cg2402*

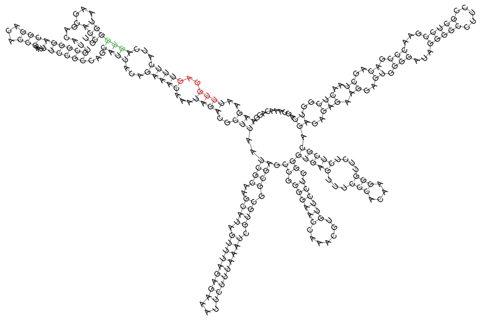

Supplement: Additional file 3: Figure S2 — Examples of secondary structures for Rfam predicted 5′-UTRs in C. glutamicum. Structures were predicted using minimum free energy and the partition function in RNAfold provided by the Vienna RNA web server[51]. The initiation codon is highlighted in green, the possible RBS in red. [file 1471-2164-14-888-S3.pdf]
